# Are social inequalities in early childhood smoking initiation explained by exposure to adult smoking? Findings from the UK Millennium Cohort Study

**DOI:** 10.1371/journal.pone.0178633

**Published:** 2017-06-02

**Authors:** David C. Taylor-Robinson, Sophie Wickham, Melisa Campbell, Jude Robinson, Anna Pearce, Ben Barr

**Affiliations:** 1 Department of Public Health and Policy, Farr Institute, University of Liverpool, Liverpool, United Kingdom; 2 Sociology, Social Policy and Criminology, University of Liverpool, Liverpool, United Kingdom; 3 UCL Institute of Child Health, London, United Kingdom; TNO, NETHERLANDS

## Abstract

**Introduction:**

To assess the socio-economic gradient in early smoking initiation at age 11 years and the extent to which any inequality was explained after accounting for longitudinal exposure to adult smoking.

**Methods:**

Analysis of the UK Millennium Cohort Study, based on 9, 609 children from ages 9 months to 11 years. The outcome was smoking initiation by age 11. Odds ratios (ORs) for smoking initiation were estimated using logistic regression, according to maternal education, whilst adjusting for baseline demographic factors. Longitudinal exposure to a regular smoker in the same room was assessed as potential mediator of the association between maternal education and early smoking, along with other socially patterned risk factors for early smoking initiation, such as parental separation and mental health.

**Results:**

Overall 2.7% (95% CI: 2.3–3.1) of children had tried a cigarette by age eleven. Children of mothers with no qualifications were more than six times as likely to have tried a cigarette than children of mothers with degree level qualifications or higher (OR 6.0 [95%CI 3.5–10.1]), with clear social gradient. Controlling for potentially mediating variables, particularly exposure to a regular adult smoker reduced the OR smoking initiation in children of mothers with no qualifications by 63% (aOR 2.9 [95%CI 1.7 to 5.1]).

**Conclusions:**

Smoking initiation is more common in disadvantaged children, and this is largely explained by regular exposure to an adult smoker in the same room. Reducing adult smoking in front of children may reduce inequalities in smoking initiation in children by over a half.

## Introduction

Early smoking initiation in children is an important determinant of long-term tobacco dependency, ever stopping smoking and risk of adverse adult health. [1] Early onset of smoking is also associated with other risky behaviours such as substance and alcohol misuse and development of significant negative social outcomes over the life course. [2] There is currently little data on very early smoking initiation. In the latest English data (national survey of six thousand 11–15 year old school children in 2014) around 18% of 11 to 15 year olds said that they had smoked at least once. [3]

There are significant social inequalities in smoking initiation in children.[4–6] Socioeconomic disadvantage is associated with higher initiation rates, particularly at younger ages, and with higher rates of transition to daily smoking. In the UK smoking uptake is reducing as a result of tobacco control, and more young people are quitting in early adolescence. Socioeconomic inequalities in smoking remain however, driven in part due to the social patterning of initiation. [4]

Exposure to adult smokers is an important risk factor for earlier initiation of childhood smoking. [7] Adult smoking influences child and adolescent smoking uptake, and is thus a key mechanism for the transmission of smoking behaviours, and potentially health inequalities, from one generation to another.[1,8] A recent systematic review identified exposure to smokers in the family (parental and sibling smoking) as significant and avoidable risk factors for uptake by children and young people. However, this study was limited to household members and did not consider how other adult smoking over time may also influence smoking uptake in children. Although many studies have assessed risk factors for early smoking initiation, [9] [1] no studies have explored the extent to which exposure to adult household smoking patterns explain inequalities in early smoking in children in a representative UK population.

A recent systematic review highlighted the need to strengthen the evidence base for equitable tobacco control interventions and policies.[10] A better understanding of the role of exposure to adult smoking patterns in mediating the relationship between socioeconomic conditions (SECs) in childhood and smoking onset is imperative in order to develop interventions to reduce the intergenerational transfer of smoking, and to reduce health inequalities.[4,11] The aim of this study was therefore to assess the socio-economic gradient in very early smoking initiation (in a nationally representative sample of children in their final year of primary school, five-six years after the UK smoking bans were introduced). We explored risk factors for early initiation of smoking; and assessed the extent to which any inequality in early smoking was attenuated after accounting for these risk factors, with a focus on longitudinal exposure to household adult smokers.

## Materials and methods

### Design, setting, and data source

We used data on children in sweeps of the Millennium Cohort Study (MCS) study conducted at 9 months 3, 5, 7 and 11 years, capturing data on around 70% of the original sample (18552 children) at age 11 years. The Millennium Cohort Study (MCS) is a nationally representative sample of children born in the UK between September 2000 and January 2002. Interviews were carried out with the main respondent, usually the mother, at each sweep, and data was also collected from a cohort child questionnaire at age 11 years. The study over-sampled children living in disadvantaged areas and those with high proportions of ethnic minority groups by means of stratified cluster sampling design. Further information on the cohort and sampling design can be found in the cohort profile. [12]. The analysis did not require additional ethical approval.

### Outcome measure: Initiation of smoking at age 11

At 11 years trained investigators asked the children whether they had “ever tried a cigarette, even if it was only a single puff?” (coded as yes/no). This question thus captures smoking initiation as outlined in Flay’s conceptual model [13]

### Exposures of interest

The primary exposure of interest was maternal academic qualifications used as a fixed measure of SEC at birth of the MCS child. The highest qualification attained by the mother was established by questionnaire at the first wave, categorized in this study by six levels: degree plus (higher degree and first degree qualifications), diploma (in higher education), A-levels (a qualification offered for high school and pre-university students), GCSE (typically taken at age 16 years) grades A*-C, GCSE grades D-G, and none of these qualifications. The main putative mediator of interest (of the association between SECs and smoking initiation) was exposure to adult smoking in the same room. The latter was captured by questions in MCS waves 1–4 which asked whether anyone regularly smoked in front of their child (in the same room): “Including yourself, does anyone smoke in the same room as [^Cohort child's name] nowadays?”. Other pre-specified risk factors in the MCS potentially associated with early childhood smoking included: sex, ethnicity (coded as white or non-white to avoid small cell counts for smoking initiation, the latter including mixed, Indian, Pakistani, Bangladeshi, black, other ethnic group), maternal age at birth of cohort child, maternal mental health problems (coded as yes if the respondent ever answered yes to the question “Has a doctor ever told you that you suffer from depression or serious anxiety?” which was asked at each wave including at 11 years) and parental separation/divorce (coded as ever reported separation or divorce at any wave up to 11 years, yes/no). [2,14,15] Maternal mental health problems and parental separation were considered as potential alternative mediators of the relationship between childhood SECs and early smoking initiation.

### Analysis strategy

We first explored the unadjusted association between maternal qualifications (primary exposure) and childhood smoking at 11 years. We then explored the associations between the other risk factors and smoking at 11 years, calculating unadjusted odds ratios (ORs) using logistic regression. We then fitted sequential adjusted models, adjusted for baseline demographic factors (sex, ethnicity, and maternal age at MCS birth), calculating adjusted ORs using for smoking on the basis of maternal qualification (with children of mothers with highest qualifications as the reference group). We first added parental separation/divorce and maternal mental health problems as potential mediators, and then in the final model we added the regular exposure to adult smoking variable. Any observed change in ORs on the addition of risk factors in a final complete case sample was taken to indicate potential mediation. [16] We estimated the change in ORs comparing mothers with the highest qualifications to those with the lowest (the SEC gap). This was calculated as 100x(OR-aOR)/(OR-1) [17,18] Analyses were conducted in Stata/SE V.13 (Stata Corporation). Sampling and response weights were used in all analyses to account for the sampling design and attrition to age 11.

We undertook a range of sensitivity analyses to explore the key findings, first repeating the analysis with equivalised household income quintile as an alternative measure of SEC. We repeated the analysis using an alternative measure of exposure to smoking (using longitudinal current parent/carer smoking status). We undertook a stratified analysis exploring the relationship between SECs and childhood smoking in families where children were never exposed to anyone smoking. We did a mediation analysis using the counterfactual framework to assess how much of the effect of SEC on smoking initiation is mediated via parental smoking (natural indirect effect). To do this we assessed the effect of a change in the level of exposure to adult smoking from the value realized under the low socio-economic condition to the value realized under the high socio-economic condition.[19] We further repeated the analysis adjusting for cumulative measures of parental separation and maternal mental health.

## Results

**Fig 1:**
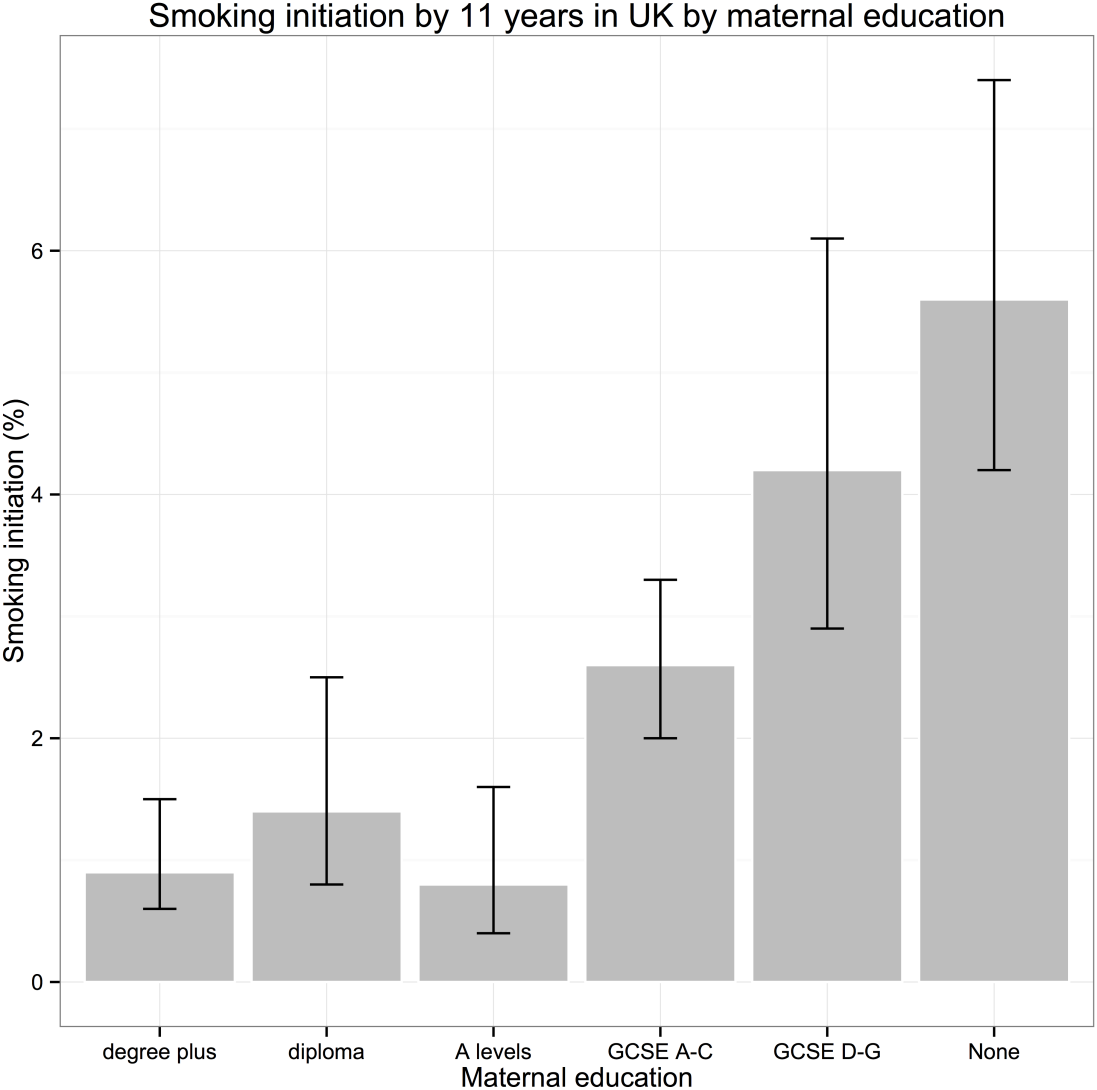
Initiation of early smoking and peer smoking at age 11 by SECs in UK children.

12, 605 children who participated in sweeps of the study at ages 9 months to 11 years had data on smoking at age 11 years. Data on cohort member smoking (main outcome) and maternal education (main exposure) were available for 12,215 participants. 9,609 (79%) had full data on all exposures of interest in the fully adjusted model. The percentage of children initiating smoking by 11 years was 2.7% (95% CI: 2.3–3.1) in the analysis sample (Table 1, Fig 1). There was a clear social gradient, with prevalence increasing in a stepwise fashion on the basis of maternal education (as a measure of SECs) from 0.9% (95% CI: 0.6–1.5) in the highest maternal qualification group (degree plus) to 5.6% (95% CI: 4.2–3.1) in children whose mother had no qualifications (Fig 1). All the other covariates of interest, except for sex, varied by level of maternal qualifications, with higher levels of teenage parenthood, non-white ethnicity, parental separation, parental mental health problems in children of mothers with lower levels of educational qualifications.

**Table 1:** Characteristics of the study population, by level of maternal academic qualification at birth of child (n = 9, 609).

	Degree plus	Diploma	A levels	GCSE	GCSE	None	Total	p-value
				A-C	D-G			
	%	%	%	%	%	%	%	
**Subjects n (%)**	1980 (20.6)	960	1,041	3305	945	1378	9609	
		-10	-10.8	-34.4	-9.8	-14.4	-100	
**Child ever tried cigarette by 11**	0.9	1.4	0.8	2.6	4.2	5.6	2.7	<0.001
**Child's sex**								
Male	50.7	52	50.6	51.8	52	51.4	51.5	
Female	49.3	48	49.4	48.2	48	48.6	48.5	0.588
**Child ethnicity**								
White	88.9	90	89.7	92.1	91.6	73.2	87.8	
Non- white	11.1	10	10.3	7.9	8.4	26.8	12.2	<0.001
**Maternal age at MCS birth**								
14–19	0.1	0.6	4.2	8.7	17.8	14.9	8.2	
20–24	2.8	9.4	15.3	19.2	25.8	26.2	17.2	
25–29	25.3	33.8	31.1	29.1	30.5	26.5	28.8	
30–34	42.7	37.3	31.6	28.6	18.4	20.6	29.4	
35+	29	19	17.8	14.4	7.5	12	16.3	<0.001
**Parent ever separated**								
No	85.7	79.2	77.9	75.6	77.5	73.3	77.6	
Yes	14.3	20.8	22.1	24.4	22.5	26.7	22.4	<0.001
**Maternal mental health problem**								
No	67.4	57.8	58.2	49.2	44.7	43.2	52.3	
Yes	32.6	42.2	41.8	50.8	55.3	56.8	47.7	<0.001
**Child exposed to smoking**								
No, never	92.2	85.8	79.2	68.6	55.5	47.2	92.2	
In at least 1 sweep	5.1	7.4	12.2	14.6	15.2	19.4	5.1	
In 2 sweeps	1.6	3.3	4.9	7.9	12.1	13.4	1.6	
In 3 sweeps	0.7	2.5	2.4	4.9	10.2	11.8	0.7	
In all sweeps up to age 11	0.4	0.9	1.3	4	7	8.3	0.4	<0.001

Missing data as follows (N): sex = 408, ethnicity = 1473, maternal age at cohort birth = 420, regular exposure to adult smoking = 2583.

### Associations of covariates with smoking initiation

**Fig 2:**
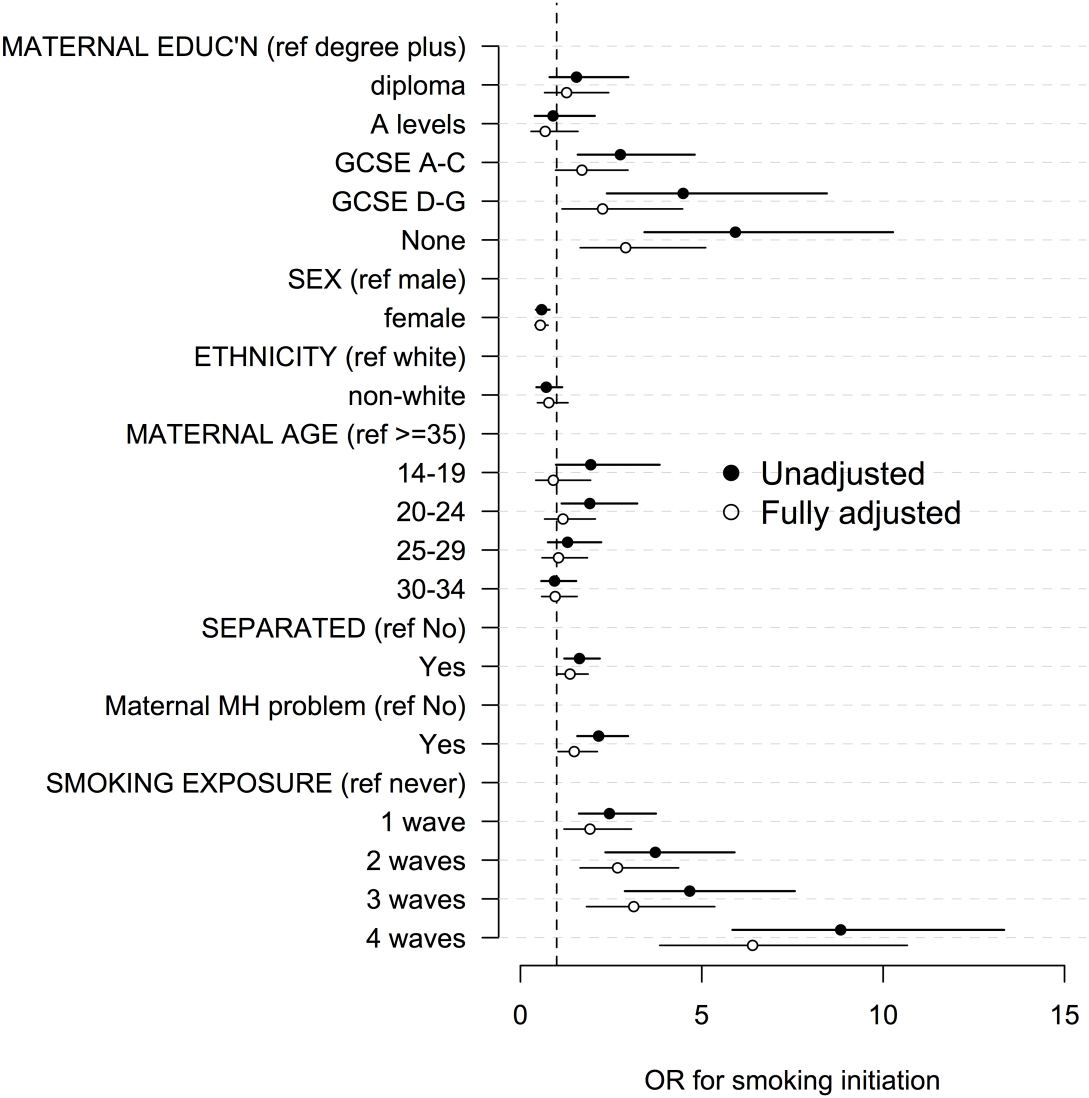
Univariate and fully adjusted associations (odds ratios, ORs) between covariates and smoking initiation at age 11 years.

In the univariate regression lower maternal education, male sex, younger maternal age at MCS birth, parental separation, maternal mental health problem diagnosis, exposure to adult smoking in the same room were associated with an increased OR for smoking initiation in children at age 11 (Table 2)(Fig 2). There was a clear social gradient, whereby the risk of smoking initiation increased as maternal educational qualifications decreased; children of mothers with no qualifications were more than five times as likely to have tried a cigarette than children of mothers with degree level qualifications or higher (OR 5.9 [95%CI 3.4–10.3]). There was a dose-response relationship between number of study waves at which adults were reported as regularly smoking in front of the cohort child, with the odds of smoking initiation reaching 8.8 (5.9–13.3) in children whose parents reported anyone regularly smoking in front of their child at all four preceding waves.

**Table 2:** Prevalence of initiation of cigarette smoking at age 11 and univariate ORs (n = 9609).

	Total %	Children tried cig %	OR	Lower, Upper CI
**Maternal education**				
Degree plus	17.8	0.9	1	1.00,1.00
Diploma	9.3	1.4	1.55	0.80,2.97
A levels	9.9	0.8	0.9	0.39,2.05
GCSE A-C	36.1	2.6	2.75	1.58,4.80
GCSE D-G	11.1	4.2	4.49	2.38,8.45
None	15.8	5.6	5.93	3.42,10.27
**Child sex**				
Male	50.3	3.4	1	1.00,1.00
Female	49.7	2	0.58	0.43,0.80
**Child ethnicity**				
White	88.1	2.8	1	1.00,1.00
Non-White	11.9	2	0.71	0.44,1.15
**Maternal age and MCS birth**				
14–19	7.3	4	1.94	0.98,3.84
20–24	16.4	4	1.91	1.14,3.22
25–29	29.1	2.7	1.3	0.76,2.23
30–34	30.1	1.9	0.94	0.58,1.54
35 and over	17	2.1	1	1.00,1.00
**Parent ever separated**				
No	77.4	2.3	1	1.00,1.00
Yes	22.6	3.8	1.63	1.21,2.19
**Maternal mental health problem**				
No	52.2	1.7	1	1.00,1.00
Yes	47.8	3.7	2.16	1.57,2.97
**Child exposed to adult smoking**				
No, never	71.1	1.4	1	1.00,1.00
In 1 sweep	12.7	3.5	2.45	1.61,3.74
In 2 sweeps	7.2	5.3	3.72	2.34,5.90
In 3 sweeps	5.1	6.6	4.67	2.88,7.57
In all sweeps up to age 9	3.9	12.6	8.83	5.85,13.34

Table 3 shows the estimates from three sequentially adjusted models. The table shows the extent to which the elevated OR of smoking initiation in 11-year-old children with mothers with no qualifications (OR 6.2 [95%CI 3.5–11.0], adjusted for demographic variables sex, ethnicity, and maternal age at MCS birth) compared to mothers with the highest qualifications attenuates when adjusting for potentially mediating variables. The OR is reduced by 20% to 5.1 (95% CI: 2.9–9.1) after adjusting for parental separation and maternal mental health diagnosis. In the final model (Fig 2), additionally adjusting for cumulative exposure to someone smoking around the child the OR is reduced by 63% comparing lowest to highest qualifications, although this remains significant (OR 2.9 95%CI 1.7 to 5.1). In the full model anyone regularly smoking in front of their child at all four preceding waves was associated with an OR of 6.4 (95% CI 3.8 to 10.7)(Fig 2).

**Table 3:** Regression models for smoking initiation at age 11. Covariate estimates using complete case analysis (n = 9,609).

		Model 1		Model 2		Model 3	
		OR (95%CI)		OR (95%CI)		OR (95%CI)	
**Maternal education**	Degree plus	1	[1.00,1.00]	1	[1.00,1.00]	1	[1.00,1.00]
	Diploma	1.47	[0.76,2.85]	1.36	[0.70,2.62]	1.27	[0.67,2.43]
	A levels	0.84	[0.37,1.93]	0.78	[0.34,1.79]	0.68	[0.29,1.59]
	GCSE A-C	2.55	[1.46,4.48]	2.24	[1.28,3.91]	1.7	[0.97,2.96]
	GCSE D-G	4.05	[2.07,7.91]	3.47	[1.77,6.78]	2.26	[1.15,4.47]
	None	6.15	[3.46,10.93]	5.12	[2.88,9.10]	2.9	[1.65,5.11]
**Child sex**	Male	1	[1.00,1.00]	1	[1.00,1.00]	1	[1.00,1.00]
	Female	0.57	[0.41,0.78]	0.57	[0.41,0.79]	0.55	[0.40,0.76]
**Child ethnicity**	White	1	[1.00,1.00]	1	[1.00,1.00]	1	[1.00,1.00]
	Non-White	0.51	[0.30,0.87]	0.56	[0.33,0.96]	0.78	[0.47,1.31]
**Maternal age and MCS birth**	14–19	1.14	[0.55,2.36]	1.15	[0.55,2.43]	0.91	[0.43,1.93]
	20–24	1.37	[0.78,2.41]	1.38	[0.78,2.43]	1.17	[0.67,2.06]
	25–29	1.09	[0.62,1.89]	1.09	[0.62,1.89]	1.05	[0.60,1.85]
	30–34	0.93	[0.56,1.54]	0.96	[0.59,1.58]	0.96	[0.59,1.56]
	35 and over	1	[1.00,1.00]	1	[1.00,1.00]	1	[1.00,1.00]
**Parent ever separated**	No			1	[1.00,1.00]	1	[1.00,1.00]
	Yes			1.44	[1.06,1.96]	1.37	[1.00,1.86]
**Maternal mental health problem**	No			1	[1.00,1.00]	1	[1.00,1.00]
	Yes			1.68	[1.18,2.38]	1.48	[1.04,2.12]
**Child exposed to adult smoking**	No, never					1	[1.00,1.00]
	In 1 sweep					1.92	[1.20,3.06]
	In 2 sweeps					2.68	[1.64,4.36]
	In 3 sweeps					3.12	[1.82,5.36]
	In all sweeps up to age 9					6.4	[3.84,10.67]

Model 1 adjusts for maternal education, sex, ethnicity and maternal age. Model 2 additionally adjusts for parental separation and maternal mental health problems. Model 3 additionally adjusts for longitudinal exposure to adult smoking.

### Sensitivity analysis

The social gradient in smoking initiation was significantly attenuated in children who were never regularly exposed to smoking, with an OR of 2.6 (95% CI 1.26 to 5.41) comparing children of mothers with no qualifications to degree plus level (S1 Fig). Using current main respondent (main carer, usually parent) smoking instead of regular exposure to any adult smoking showed a similar pattern with increasing risk the more waves the parent reported smoking with the OR of early smoking of 5.2 (3.6–7.4) in children whose parents who smoked at all waves of the study compared to those who had not smoked. The OR attenuation in model 3 was similar when we adjusted for cumulative longitudinal current parental smoking, instead of anyone regularly smoking in front of the child (S1 Table). The conclusions of the study were similar when we used household income as the measure of SEC (S2 Table). In a mediation analysis using the counterfactual framework 51% of the total effect of socio-economic conditions (low versus high) on odds of smoking at age 11 years in UK children was mediated through exposure to adult smoking in the same room (S1 File). Repeating the analysis using cumulative measures of parental separation and maternal mental health did not alter the results.

## Discussion

Using a nationally representative sample of UK children born in 2000, this study explored the association of maternal and social factors on early initiation of childhood cigarette smoking by age 11 years and found that three percent of children, who had yet to start secondary school, reported having tried a cigarette. There are stark inequalities in early smoking initiation in UK children. The odds of early initiation were about six times higher in disadvantaged children, but this was attenuated by over a half after adjusting for regular exposure to a smoker in the household. There was a clear dose response relationship with exposure to parental or other adult smoking in front of the child. Our data show that exposure to adult smoking is one of the most important mediators of inequalities in early smoking initiation, and suggest that adults who smoke in the same room as children increase their risk of early smoking initiation. Since exposure to, and viewing, adult smoking is a preventable and modifiable risk factor, policies to reduce the visibility of adult smoking to children should be a key focus for reducing health inequalities. Although influencing children’s exposure to smoking within the home is a difficult arena for policy-making any tobacco control policy that impacts on the adult smoking prevalence is likely to be helpful in that it may reduce the prevalence of parental smoking in front of children.

### Comparison with other findings

In this study we found in a UK sample of eleven year olds (who had not yet started secondary school) around 3% reported initiation of smoking. The latest survey data did not record any 11 year olds as regularly smoking, and less than 0.5% of 12 year olds were regular smokers. Thereafter, the proportions increased with age, up to 8% of 15 year olds. [3] National survey data suggests that almost a fifth (18%) of children aged 11–15 in England have tried smoking at least once [3] with around 3% smoking regularly, at least one cigarette per week. This is the lowest level recorded since the survey began in 1982, and continues the decline since 2003, when 42% of pupils reported that they had tried smoking. However, this lower figure may reflect a reluctance to report smoking for fear of intervention or experiencing stigma. [20] It is unclear how many of these children will go on to smoke regularly.

Numerous studies have identified low SES as a risk factor for smoking behavours. [21–25]. However, our study is one of the largest to explore inequalities in smoking initiation, and is the first to quantify the contribution of exposure to adult smoking behaviours in attenuating social inequalities in early smoking initiation in a nationally representative sample of 11-year-old children in the UK. Our study corroborates a smaller population-based prospective birth cohort study of almost 4000 children in Queensland, Australia found that one in six participants first smoke cigarettes at or before 14 years, and identified maternal education, smoking and marital circumstances predicted earlier onset of smoking.[2]

We found a clear dose response relationship linking exposure to parental or *other adult* smoking in front of the child and smoking initiation in 11 year old children. This corroborates a systematic review of 58 studies that demonstrated that smoking by siblings, parents or other household members, and particularly by parents, is a strong and significant influence on childhood smoking uptake. [1] The odds of uptake of smoking in children were increased if at least one parent smoked (OR 1.72, 95% CI 1.59 to 1.86), and more so if both parents smoked (OR 2.73, 95% CI 2.28 to 3.28). The authors further estimate that in England and Wales, around 17 000 young people take up smoking by the age of 15 each year as a consequence of exposure to household smoking. [1] Our data add to this analysis by showing the cumulative nature of this risk over the early life course, with children regularly exposured to adult smoking in the same room up to age 11 years being six times more likely to initiate smoking.

Green and colleagues highlight parental smoking as a mechanism particularly worthy of further exploration for addressing health inequalities in adolescent smoking. [4] Our analysis shows that exposure to adult smoking behaviour is a major contributor to inequalities in smoking initiation. The mechanism for this relationship likely reflects a combination of role modelling behaviour and potentially access to cigarettes, and highlights the importance of protecting children from the adverse effects of smoking role models. Our analysis showed the strongest effect with exposure to any regular smoker in the same room, which could be parents, siblings or other regular household contacts. The relationship with regular parental smoking was similar, but not quite as strong. This highlights the harmful impact of close observation of any adult smoking behaviour, which is likely to be increasingly concentrated in areas of inequalities. [26]

Maternal mental health and parental separation only slightly attenuated the social patterning of early childhood smoking initiation in our study. Both of these factors have been identified as risk factors for early initiation of smoking in previous studies, and are socially patterned. [2,14,27].

### Strengths and limitations

A key strength is that this study used secondary data from a large, contemporary UK cohort and the results are likely to be generalizable to other high-income countries. A wide range of information is collected in the MCS, which allowed us to explore a range of risk factors for early smoking initiation over time, including different measures of SEC. A limitation of our paper is the self-reported nature of the smoking outcome, though studies suggest that adolescent report of smoking is reasonably accurate.[28] We do not know extent to which early smoking initiation as measured in this study leads to actual smoking, thought it will be possible to assess this in future studies of MCS children. The analysis by Green et al suggests that there are inequalities in progression to daily smoking so one might expect inequalities in initiation to be larger in terms of who actually goes on to smoke.[4] It is possible that children growing up in more advantaged SECs are less likely to report smoking initiation, and generally self-report is less among teens. Missing data are a ubiquitous problem in cohort studies. A complete case analysis was used, including 78% of the eligible sample. Sampling and response weights were used in all analyses here to account for the sampling design and attrition to age 11, however these cannot account for item missingness. Complete case analyses can be inefficient, particularly in smaller datasets, or if those who were missing data are different (in terms of the associations under study) than those who were included. However, in this analysis the sample was large, and the internal associations, which were the targets of inference within the sample population, are likely to be valid. In our analysis we have implicitly followed the Baron and Kenny approach. Alternative methods for mediation analysis are continuously being developed, but have their limitations in terms of application in routinely available software.[18] We repeated a simplified version of our main analysis using a counterfactual approach, and found similar results (appendix).

### Policy and practice implications

Since most smokers start before the age of 21 years preventive efforts need to be focussed in the childhood and adolescent period.[29] It is also essential to identify interventions that can reduce overall smoking uptake, and at the same time reduce inequalities. Few studies have assessed the equity impact of tobacco control interventions.[10] A recent systematic review found that of the studies evaluating the equity impact of youth smoking interventions, more interventions/policies were found to increase inequalities that reduce them.[29] Our interpretation of these results is that policies to reduce smoking in front of children may reduce inequalities in smoking initiation in children by around half.

From a public health perspective, in order to reduce inequalities, effective policies focused on reducing smoking in parents with young children should be intensified. Many of these are defined and promoted by the WHO Framework Convention on Tobacco Control. Tobacco control programs should focus on the impact of the wider household and neighbourhood childhood environment on the onset of smoking in young smokers. Our study highlights the harmful impact of exposure to smoking role models, reinforcing the importance of smoke-free environments for all children.

Smoking remains a major vector of the intergenerational transmission of health inequalities. Whilst the long-term solution to health inequalities in smoking is likely to be one that takes broader action to address the social determinants of health our analysis provides further evidence that addressing modifiable risk factors in the childhood period can reduce inequalities across the life course.

## Supporting information

S1 Fig Experimentation with early smoking and peer smoking at age 11 by SECs in UK children never exposed to parental smoking (N = 7060). (DOCX)

S1 Table *Table 4: Alternative final model using parental smoking as a potential risk factor instead of “smoking infront of child”, complete case analysis (n = 9,667). (DOCX)

S2 Table Repeating the analysis using income as a measure of SECs. (DOCX)

S1 File Mediation analysis using counterfactual framework to assess what would be the effect of a change in the level of exposure to smoking in adults from the value realized under the low socio-economic condition to the value realized under the high socio-economic condition. (DOCX)
