# Supplementary material for: Are social inequalities in early childhood smoking initiation explained by exposure to adult smoking? Findings from the UK Millennium Cohort Study
Source: PLoS One. 2017 Jun 2;12(6):e0178633. doi: 10.1371/journal.pone.0178633 (PMC5456267; doi:10.1371/journal.pone.0178633)
Supplement: S1 Fig — (DOCX) [file pone.0178633.s001.docx]

**S1 fig: Experimentation with early smoking and peer smoking at age 11 by SECs in UK children never exposed to parental smoking (N=7060)**
