# Supplementary material for: Are social inequalities in early childhood smoking initiation explained by exposure to adult smoking? Findings from the UK Millennium Cohort Study
Source: PLoS One. 2017 Jun 2;12(6):e0178633. doi: 10.1371/journal.pone.0178633 (PMC5456267; doi:10.1371/journal.pone.0178633)
Supplement: S1 Table — (DOCX) [file pone.0178633.s002.docx]

**S1 table: *Table 4: Alternative final model using parental smoking as a potential risk factor instead of “smoking in front of child”, complete case analysis (n = 9,667)**

|  |  | **Model 4** |  |
| --- | --- | --- | --- |
|  |  | **OR (95%CI)** |  |
|  |  |  |  |
| **Maternal education** | Degree plus | 1 | [1.00,1.00] |
|  | Diploma | 1.16 | [0.62,2.18] |
|  | A levels | 0.65 | [0.28,1.52] |
|  | GCSE A-C | 1.7 | [0.97,2.99] |
|  | GCSE D-G | 2.22 | [1.09,4.55] |
|  | None | 3.01 | [1.69,5.37] |
| **Child sex** | Male | 1 | [1.00,1.00] |
|  | Female | 0.59 | [0.42,0.84] |
| **Child ethnicity** | White | 1 | [1.00,1.00] |
|  | Non-White | 0.77 | [0.47,1.26] |
| **Maternal age and MCS birth** | 14-19 | 0.85 | [0.40,1.81] |
|  | 20-24 | 1.26 | [0.74,2.17] |
|  | 25-29 | 1 | [0.58,1.72] |
|  | 30-34 | 0.89 | [0.55,1.44] |
|  | 35 and over | 1 | [1.00,1.00] |
| **Parent ever divorced/separated** | No | 1 | [1.00,1.00] |
|  | Yes | 1.39 | [1.03,1.89] |
| **Parental mental health diagnosis** | No | 1 | [1.00,1.00] |
|  | Yes | 1.37 | [0.96,1.93] |
| **Parent currently smokes cigarettes** | No, never | 1 | [1.00,1.00] |
|  | In at least 1 sweep | 1.41 | [0.68,2.94] |
|  | In 2 sweeps | 1.69 | [0.82,3.49] |
|  | In 3 sweeps | 1.41 | [0.66,3.03] |
|  | In 4 sweeps | 2.68 | [1.63,4.38] |
|  | In all 5 sweeps | 3.13 | [2.13,4.61] |
