# Supplementary material for: Are social inequalities in early childhood smoking initiation explained by exposure to adult smoking? Findings from the UK Millennium Cohort Study
Source: PLoS One. 2017 Jun 2;12(6):e0178633. doi: 10.1371/journal.pone.0178633 (PMC5456267; doi:10.1371/journal.pone.0178633)
Supplement: S2 Table — (DOCX) [file pone.0178633.s003.docx]

**S2 Table. Repeating the analysis using income as a measure of SECs**

|  |  | **Model 1** |  | **Model 2** |  | **Model 3** |  | **Model 4** |  |
| --- | --- | --- | --- | --- | --- | --- | --- | --- | --- |
|  |  | **OR (95%CI)** |  | **OR (95%CI)** |  | **OR (95%CI)** |  | **OR (95%CI)** |  |
|  |  |  |  |  |  |  |  |  |  |
| **Income quintile** | highest quintile | 1 | [1.00,1.00] | 1 | [1.00,1.00] | 1 | [1.00,1.00] | 1 | [1.00,1.00] |
|  | fourth quintile | 1.04 | [0.53,2.05] | 1.02 | [0.51,2.02] | 0.96 | [0.49,1.90] | 0.9 | [0.45,1.80] |
|  | third quintile | 1.94 | [1.17,3.23] | 1.92 | [1.14,3.25] | 1.7 | [1.01,2.86] | 1.41 | [0.84,2.37] |
|  | second quintile | 3.52 | [1.98,6.27] | 3.65 | [2.05,6.52] | 3.06 | [1.69,5.56] | 2.02 | [1.06,3.86] |
|  | lowest quintile | 4.97 | [2.95,8.37] | 5.6 | [3.14,10.00] | 4.61 | [2.58,8.23] | 2.6 | [1.35,4.99] |
| **Child sex** | Male |  |  | 1 | [1.00,1.00] | 1 | [1.00,1.00] | 1 | [1.00,1.00] |
|  | Female |  |  | 0.55 | [0.40,0.77] | 0.56 | [0.40,0.77] | 0.55 | [0.39,0.76] |
| **Child ethnicity** | White |  |  | 1 | [1.00,1.00] | 1 | [1.00,1.00] | 1 | [1.00,1.00] |
|  | Non-White |  |  | 0.45 | [0.28,0.74] | 0.5 | [0.30,0.83] | 0.7 | [0.42,1.15] |
| **Maternal age** | 14-19 |  |  | 0.85 | [0.38,1.88] | 0.9 | [0.40,1.99] | 0.79 | [0.36,1.73] |
|  | 20-24 |  |  | 1.18 | [0.66,2.08] | 1.21 | [0.69,2.13] | 1.1 | [0.63,1.93] |
|  | 25-29 |  |  | 1.02 | [0.58,1.79] | 1.02 | [0.58,1.81] | 1.01 | [0.57,1.80] |
|  | 30-34 |  |  | 0.99 | [0.60,1.64] | 1.01 | [0.62,1.67] | 1 | [0.61,1.65] |
|  | 35 and over |  |  | 1 | [1.00,1.00] | 1 | [1.00,1.00] | 1 | [1.00,1.00] |
| **Parent ever divorced/separated** | No |  |  |  |  | 1 | [1.00,1.00] | 1 | [1.00,1.00] |
|  | Yes |  |  |  |  | 1.36 | [1.00,1.85] | 1.31 | [0.96,1.80] |
| **Parental mental health diagnosis** | No |  |  |  |  | 1 | [1.00,1.00] | 1 | [1.00,1.00] |
|  | Yes |  |  |  |  | 1.58 | [1.11,2.25] | 1.43 | [1.00,2.05] |
| **Child exposed to adult smoking** | No, never |  |  |  |  |  |  | 1 | [1.00,1.00] |
|  | In 1 sweep |  |  |  |  |  |  | 1.91 | [1.19,3.07] |
|  | In 2 sweeps |  |  |  |  |  |  | 2.75 | [1.69,4.48] |
|  | In 3 sweeps |  |  |  |  |  |  | 2.97 | [1.66,5.29] |
|  | In all sweeps up to age 9 |  |  |  |  |  |  | 6.3 | [3.59,11.07] |
