# Supplementary material for: Are social inequalities in early childhood smoking initiation explained by exposure to adult smoking? Findings from the UK Millennium Cohort Study
Source: PLoS One. 2017 Jun 2;12(6):e0178633. doi: 10.1371/journal.pone.0178633 (PMC5456267; doi:10.1371/journal.pone.0178633)
Supplement: S1 File — (DOCX) [file pone.0178633.s004.docx]

**S1 File. Mediation analysis using counterfactual framework to assess what would be the effect of a change in the level of exposure to smoking in adults from the value realized under the low socio-economic condition to the value realized under the high socio-economic condition**

The figure below shows the directed acyclic graph (DAG) for a causal mediation analysis using the counterfactual framework. We estimate the total effect of the exposure (high versus low socio-economic conditions – coded as GCSE C or higher (yes/no)) on smoking at age 11 years when we change the exposure from level *a** to *a*, given by E[Y_a_-Y_a*_] after accounting for potential confounding by known covariates as illustrated in the DAG*.*

Using the paramed package in STATA (developed by Richard Emsley), we calculated the natural direct effect and indirect effects. The natural indirect effect (NIE), formally defined by E[Y_a_M_a_-Y_a_M_a*_] answers the counterfactual question: if we were to hold socio-economic conditions constant at the higher socio-economic condition, what would be the effect of a change in the level of exposure to smoking in adults from the value realized under the low socio-economic condition to the value realized under the high socio-economic condition? The total effect of low (versus high) socio-economic conditions on smoking in children was OR 2.2 (bootstrap 95%CI 1.6-3.0) with a NIE of 1.37 (bootstrap 95%CI 1.3-1.5)

Overall 51% of the total effect of socio-economic conditions (low versus high) on odds of smoking at age 11 years in UK children is mediated through exposure to adult smoking. For the mediation analysis to have a causal interpretation, we assume no exposure/mediator interaction; that adjustment for the four types of confounding has been addressed and that there is no post-treatment confounding. The four types of confounding are: (1) confounding of the exposure-outcome relationship; (2) confounding of the mediator-outcome relationship; (3) confounding of the exposure-mediator association; and (4) mediator-outcome confounders also affected by the exposure. For controlled direct effect, assumptions (1) and (2) are required. For the identification of natural direct and indirect effects, assumptions (3) and (4) are also needed.

**Directed acyclic graph in Daggity for mediation analysis**

**
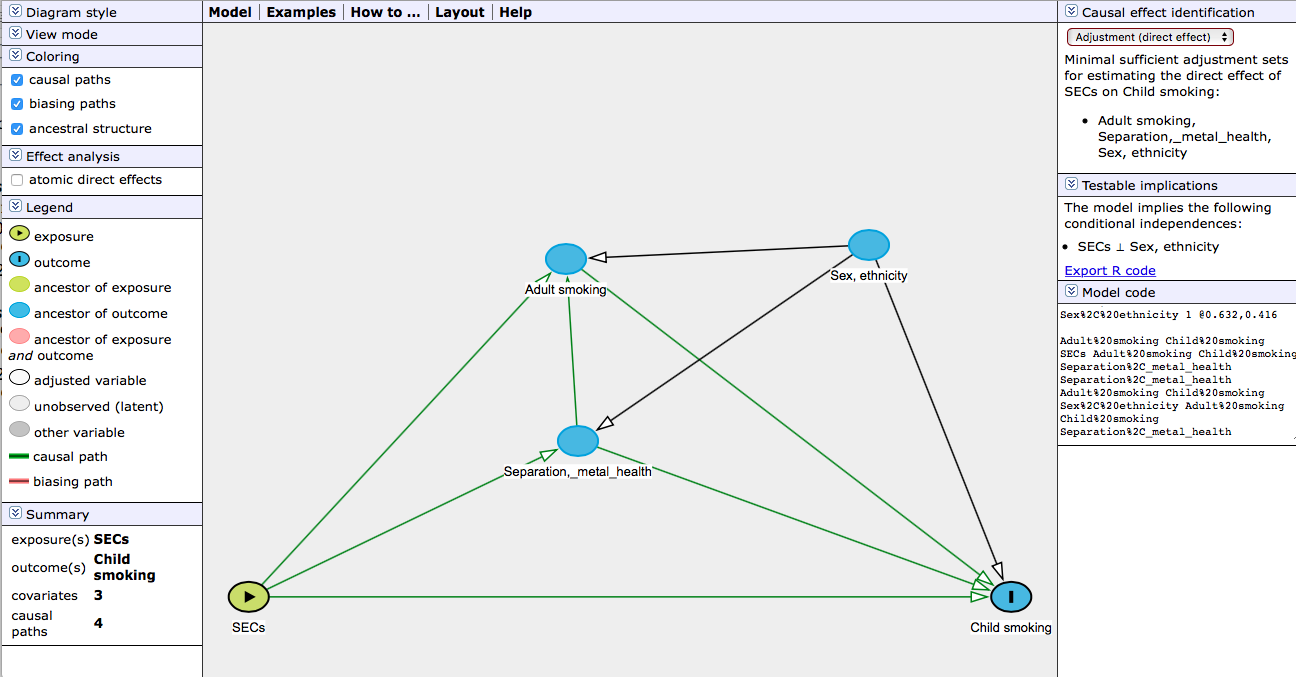
**

**Model code:**

Adult%20smoking 1 @-0.004,0.453

Child%20smoking O @0.930,1.341

SECs E @-0.669,1.341

Separation%2C_metal_health 1 @0.021,0.931

Sex%2C%20ethnicity 1 @0.632,0.416

Adult%20smoking Child%20smoking

SECs Adult%20smoking Child%20smoking Separation%2C_metal_health

Separation%2C_metal_health Adult%20smoking Child%20smoking

Sex%2C%20ethnicity Adult%20smoking Child%20smoking Separation%2C_metal_health

**Paramed STATA output**

paramed puff, avar(amated1bin_slw) mvar(persistsmoke) cvars(ahcsexa0_spm ethnic_bin ageatbirthcat

> matmentalhealth everdiv) a0(0) a1(1) m(6) yreg(logistic) mreg(linear) nointer boot reps(500) see

> d(1234)

Iteration 0: log likelihood = -1066.5767

Iteration 1: log likelihood = -978.2306

Iteration 2: log likelihood = -964.0986

Iteration 3: log likelihood = -963.52273

Iteration 4: log likelihood = -963.5212

Iteration 5: log likelihood = -963.5212

Logistic regression Number of obs = 9588

LR chi2(7) = 206.11

Prob > chi2 = 0.0000

Log likelihood = -963.5212 Pseudo R2 = 0.0966

------------------------------------------------------------------------------

puff | Coef. Std. Err. z P>|z| [95% Conf. Interval]

-------------+----------------------------------------------------------------

amated1bin~w | .4573788 .1492364 3.06 0.002 .1648808 .7498768

persistsmoke | .4187034 .0416541 10.05 0.000 .3370629 .5003439

ahcsexa0_spm | -.5616457 .1413247 -3.97 0.000 -.838637 -.2846545

ethnic_bin | .0105384 .2263288 0.05 0.963 -.4330578 .4541346

ageatbirth~t | -.0650559 .0602553 -1.08 0.280 -.1831541 .0530422

matmentalh~h | .4784994 .1497485 3.20 0.001 .1849976 .7720012

everdiv | .4578936 .1485041 3.08 0.002 .166831 .7489563

_cons | -4.412683 .3386448 -13.03 0.000 -5.076415 -3.748952

------------------------------------------------------------------------------

Source | SS df MS Number of obs = 10921

-------------+------------------------------ F( 6, 10914) = 336.78

Model | 2771.86624 6 461.977707 Prob > F = 0.0000

Residual | 14971.4065 10914 1.37176164 R-squared = 0.1562

-------------+------------------------------ Adj R-squared = 0.1558

Total | 17743.2728 10920 1.62484183 Root MSE = 1.1712

---------------------------------------------------------------------------------

persistsmoke | Coef. Std. Err. t P>|t| [95% Conf. Interval]

----------------+----------------------------------------------------------------

amated1bin_slw | .7666209 .0267962 28.61 0.000 .7140955 .8191464

ahcsexa0_spm | .0070909 .0224172 0.32 0.752 -.036851 .0510327

ethnic_bin | -.5226536 .0338292 -15.45 0.000 -.588965 -.4563423

ageatbirthcat | -.1885059 .0101264 -18.62 0.000 -.2083554 -.1686564

matmentalhealth | .3137635 .0230349 13.62 0.000 .2686108 .3589162

everdiv | .1755831 .0276811 6.34 0.000 .1213232 .229843

_cons | 1.411664 .0509901 27.69 0.000 1.311714 1.511614

---------------------------------------------------------------------------------

| Estimate Std Err P>|z| [95% Conf Interval]

-------------+-------------------------------------------------------

cde | 1.5799272 .14923639 0.002 1.1792463 2.1167505

nie | 1.3784874 .03384658 0.000 1.2900068 1.4730367

te | 2.1779097 .14375027 0.000 1.6431499 2.8867061

cde:controlled direct effect, nie:natural indirect effect, te:total effect

------------------------------------------------------------------------------

| Observed Bootstrap

| Coef. Bias Std. Err. [95% Conf. Interval]

-------------+----------------------------------------------------------------

cde | 1.5799272 .0190479 .25742235 1.147808 2.147193 (BC)

nie | 1.3784874 -.010879 .04683745 1.303123 1.480714 (BC)

te | 2.1779097 .0047315 .33252769 1.599168 2.967366 (BC)

------------------------------------------------------------------------------

(BC) bias-corrected confidence interval

(BC) bias-corrected confidence interval

Calculation of proportion mediated (see P48 of TVW Explanation in Causal Inference

https://global.oup.com/academic/product/explanation-in-causal-inference-9780199325870?cc=gb&lang=en&)

> NIE <- 1.378

> NDE <- 1.5799

>

> (NDE*(NIE-1)) / (NDE*NIE -1)

[1] 0.5073495
